# Intracoronary fixed dose of nitroprusside via thrombus aspiration catheter for the prevention of the no-reflow phenomenon following primary percutaneous coronary intervention in acute myocardial infarction

**DOI:** 10.3892/etm.2013.1139

**Published:** 2013-06-04

**Authors:** YU-JUN ZHAO, XIANG-HUA FU, XIAO-XIAO MA, DONG-YING WANG, QIU-LI DONG, YAN-BO WANG, WEI LI, KUN XING, XIN-SHUN GU, YUN-FA JIANG

**Affiliations:** 1 Department of Cardiology, Second Hospital of Hebei Medical University, Shijiazhuang, Hebei 050000;; 2 Hebei Medical University North China Petroleum Bureau General Hospital, Renqiu, Hebei 062552, P.R. China

**Keywords:** thrombus aspiration, tirofiban, no-reflow, ST elevation myocardial infarction, nitroprusside

## Abstract

Previous studies have shown that intracoronary (IC) nitroprusside (NTP) injection is a safe and effective strategy for the treatment of no-reflow (NR) during percutaneous coronary intervention (PCI). The present study tested the hypothesis that, on the basis of thrombus aspiration for the treatment of ST-segment elevation myocardial infarction (STEMI), the selective IC administration of a fixed dose of NTP (100 *μ*g) plus tirofiban is a safe and superior treatment method compared with the IC administration of tirofiban alone for the prevention of NR during primary PCI. A total of 162 consecutive patients with STEMI, who underwent primary PCI within 12 h of onset, were randomly assigned to two groups: Group A, IC administration of a fixed dose of NTP (100 *μ*g) plus tirofiban (10 *μ*g/kg) and group B, IC administration of tirofiban (10 *μ*g/kg) alone (n=80 and n=82, respectively). The drugs were selectively injected into the infarct-related artery (IRA) via a thrombus aspiration catheter advanced into the IRA. The primary end-point was post-procedural corrected thrombolysis in myocardial infarction (TIMI) frame count (CTFC). The proportion of complete (>70%) ST-segment resolution (STR); the TIMI myocardial perfusion grade (TMPG) 2–3 ratio following PCI; the peak value of creatine kinase (CK)-MB; the TIMI flow grade; the incidence of major adverse cardiac events (MACEs) and the left ventricular ejection fraction (LVEF) after 6 months of follow-up were observed as the secondary end-points. There were no significant differences in the baseline clinical and angiographic characteristics between the two groups. Compared with group B, group A had i) a lower CTFC (23±7 versus 29±11, P=0.000); ii) a higher proportion of complete STR (72.5 versus 55.9%, P=0.040); iii) an enhanced TMPG 2–3 ratio (71.3 versus 53.7%, P=0.030) and iv) a lower peak CK-MB value (170±56 versus 210±48 U/l, P=0.010). There were no statistically significant differences in the final TIMI grade-3 flow between the two groups (92.5 versus 91.5% for groups A and B, respectively; P=0.956). The LVEF at 6 months was higher in group A than group B (63±9 versus 53±11%, respectively; P=0.001); however, the incidence of MACEs was not statistically different between the two groups, although there was a trend indicating improvement in group A (log rank χ^2^=0.953, P=0.489). The selective IC administration of a fixed dose of NTP (100 *μ*g) plus tirofiban via a thrombus aspiration catheter advanced into the IRA is a safe and superior treatment method compared with tirofiban alone in patients with STEMI undergoing primary PCI. This novel therapeutic strategy improves the myocardial level perfusion, in addition to reducing the infarct size. Furthermore, it may improve the postoperative clinical prognosis following PCI.

## Introduction

Primary percutaneous coronary intervention (PCI) significantly improves the survival of patients with ST-segment elevation myocardial infarction (STEMI) (1). However, post-procedural microvascular obstruction, despite the presence of normal epicardial flow, remains an important limitation of the procedure, which substantially reduces the beneficial effects of PCI (2,3). This complication occurs in 0.6–5% of elective PCIs but it may be observed in up to 50% of primary PCI cases, involving the treatment of thrombus-containing lesions (4).

An optimal standard treatment for no-reflow (NR) has not yet been established. Based on the multifactorial pathogenesis of NR during STEMI, a combination of mechanical and pharmacological approaches appears to offer an enhanced solution for achieving the desired microvascular reperfusion.

Thrombus-aspiration-assisted primary intervention achieves complete ST-segment resolution (STR) in only 60% of patients (5). Importantly, the administration of current anti-platelet therapies during reperfusion therapy for STEMI has not eliminated the NR phenomenon (6). Nitroprusside (NTP) is an alternative drug that is, at present, being used for the reversal of the NR phenomenon. A study investigating the use of this agent in the treatment of the NR phenomenon revealed some promising preliminary results (7).

To date, to the best of our knowledge, there have been no investigations into the combined strategy of thrombus aspiration and tirofiban plus NTP. Thus, the aim of the present prospective randomized control study was to assess the effect of NTP in combination with the therapy currently considered the most efficacious for STEMI, that is, thrombus aspiration and tirofiban, in the prevention of NR.

## Patients and methods

### Study population

From January 2010 to December 2012, all consecutive patients with STEMI occurring within 12 h of the onset of symptoms who underwent emergency PCI in the Department of Cardiology of the Second Hospital of Hebei Medical University (Shijiazhuang, China) were enrolled in the study.

Inclusion criteria: i) Acute STEMI was diagnosed on the basis of typical chest pain lasting >30 min; ii) ST-elevation of ≥1 mm in at least two contiguous electrocardiogram (ECG) leads and/or ≥2 mm in the precordial leads; iii) thrombolysis in myocardial infarction (TIMI) flow ≤2 at baseline angiography.

The exclusion criteria were: i) An age of >75 years; ii) cardiogenic shock (defined as a systolic blood pressure of <90 mmHg for >30 min or the requirement for intravenous pressors or intra-aortic balloon counter pulsation); iii) a history of bleeding diathesis; iv) major surgery during the previous 6 weeks; v) gastrointestinal or genitourinary bleeding in the preceding 6 months; vi) a cerebrovascular event in the previous year; vii) a platelet count of <100,000/mm^3^; viii) renal insufficiency, defined as a serum creatinine level of >2.5 mg/dl; ix) chronic hemodialysis or pregnancy; x) rescue intervention following failed thrombolysis; xi) contraindications to aspirin or clopidogrel; xii) inability to provide informed consent.

### Procedure

Following diagnostic coronary angiography, patients who met the eligibility criteria were assigned in a 1:1 ratio into two groups. Subsequent to wire crossing, thrombus aspiration was performed. A ZEEK aspiration thrombectomy catheter (Zeon Medical Inc., Tokyo, Japan) was removed from the body, flushed with heparinized saline and subsequently reintroduced into the culprit vessel beyond the occlusion site, prior to the selective administration of intracoronary (IC) drugs. The drugs were infused into the infarct-related artery (IRA) only if the distal vessels were visualized following aspiration. Conversely, balloon predilatation was permitted if thrombus aspiration was ineffective. At this stage, prior to the coronary stent implantation, the patient was randomly assigned either to group A (100 *μ*g NTP as a fast bolus, followed by 10 *μ*g/kg tirofiban, administered in 20 ml heparinized saline in 2 min as a slow bolus) or group B (5 ml heparinized saline as a fast bolus followed by 10 *μ*g/kg tirofiban, administered in 20 ml heparinized saline in 2 min as a slow bolus). In addition, the patients in the two groups received a 12-h intravenous infusion of 0.15 *μ*g/kg/min tirofiban following angioplasty. If it was not possible to advance the thrombus aspiration device in the culprit vessel for mechanical reasons, drug administration was performed through a guiding catheter following predilatation. Patients subject to this method of administration were included in the overall and the subgroup analyses. The data were analyzed according to the intention-to-treat principle.

Following the injection, the surgeon proceeded with the angioplasty in the usual fashion. Multiple types of balloons and drug-eluting stents were used. For NR occurring during the subsequent stages of the angioplasty, the surgeons used any drug other than NTP. The ECG and blood pressure were monitored during the procedure. Following the procedure, patients in either group were treated with standard therapies for coronary heart disease, including aspirin, clopidogrel, fluvastatin calcium, nitrate esters, β-receptor antagonists and angiotensin converting enzyme inhibitor/angiotensin II receptor blockers. All patients were orally pretreated with 300 mg aspirin and 300 mg clopidogrel prior to the procedure. The study was approved by the ethics committee of the Second Hospital of Hebei Medical University, and each patient provided written, informed consent.

For each patient, routine demographic and clinical data, procedural results and in-hospital complications were prospectively entered into a computerized databank. All data were verified by a retrospective review of the patient records.

### Study end-points

The following were the primary end-points of the study: i) Coronary blood flow in the IRA subsequent to angioplasty, as determined by the corrected TIMI frame count (CTFC) (8); ii) the proportion of patients with TIMI myocardial perfusion grade (TMPG) 2–3 following the procedure (9); iii) the proportion of patients with complete STR at 90 min subsequent to the intervention. CTFCs and MBG were assessed by two experienced surgeons who were blinded to the study medications according to standardized methods. The qualifying cine run was the first one obtained following satisfactory relief of the epicardial culprit stenosis. STR was measured immediately subsequent to the angioplasty using a 12-lead ECG tracing by a separate investigator who was blinded to the angiographic data. Complete STR was defined as >70% reduction in the ST-segment elevation of the lead with the highest elevation on admission.

Secondary end-points included the proportion of patients with TIMI grade-3 (10) flow following the procedure, left ventricular ejection fraction (LVEF) and the combined rate of cardiac death, reinfarction and target-vessel revascularization at 180 days.

### Statistical analysis

Sample size was calculated for the first primary end-point. To detect a difference in CTFC of 10 frames (11), assuming a standard deviation of 15 frames in each group with a=5% and power of 80%, 50 patients were required to be randomized to each arm. Differences in the end-points between the groups were compared using the Student’s t-test for continuous variables and the χ^2^ test for categorical data. Clinical follow-up data were obtained from the composite clinical end-point and were analyzed using Kaplan-Meier survival curve analysis, while differences between the groups were compared using the log rank test. Statistical analysis was performed using SPSS statistical software for Windows version 13.0 (SPSS, Inc., Chicago, IL, USA). P<0.05 was considered to indicate a statistically significant difference.

## Results

### Baseline clinical and angiographic characteristics

A total of 165 patients were randomized, among whom three did not undergo primary PCI (two patients underwent an emergency bypass procedure and one was excluded due to a lack of consent). A total of 162 patients were included in the per-protocol analysis.

The clinical and angiographic characteristics of the patients are presented in Table I. The age, gender distribution, incidence of coronary risk factors and recanalization time from symptom onset, as well as the IRA and pre-TIMI flow grade were similar between the two groups.

### Procedural characteristics

The PCI procedures were observed to be successful in all patients in the two groups. The post-procedural CTFC in group A was significantly lower than that in group B (23±7 versus 29±11, respectively; P=0.000), while the post-procedural TMPG 2–3 ratio in group A was significantly higher than that in group B (71.3 versus 53.7%, respectively; P=0.030). There was significant difference in the ratio of complete STR between groups A and B at 90 min subsequent to PCI. (72.5 versus 55.9%, respectively; P=0.04). However, considerable improvements in epicardial coronary flow were observed in the two groups immediately following the procedure. There were no statistically significant differences in the final TIMI-3 flow between the two groups (92.5 versus 91.5% for groups A and B, respectively; P=0.956; Table II).

No statistically significant differences were observed in stent diameter and stent length between the two groups following PCI. At discharge, there were no differences in the rate of use of aspirin, clopidogrel, β-blockers, angiotensin converting enzyme inhibitors or statins.

### Feasibility and safety

A transient systolic pressure drop below 90 mmHg occurred in three patients in group A (3.75%) and none in group B. There were no severe hemorrhage symptoms in either group. The occurrence of minor bleeding events in group A (3.75%) was not significantly higher than that in group B (6.1%; P>0.05).

### Follow-up results at 6 months

**Figure 1.**
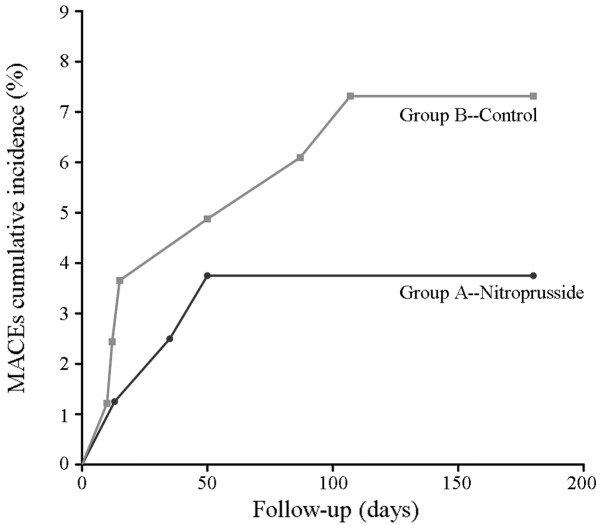
Kaplan-Meier curves of the cumulative percentage of the first event of target vessel revascularization, reinfarction or cardiac death. MACEs, major adverse cardiac events. Log-rank χ^2^=0.953, P=0.489.

By 6 months, the LVEF was higher in group A (63±9 versus 53±11 for groups A and B, respectively; P<0.01). The Kaplan-Meier analysis showed that the separation trend of the two curves appeared to continue subsequent to the 6 months. The rates of cardiac death, reinfarction and target-vessel revascularization in group A were 1.25% (1/80), 1.25% (1/80) and 1.25% (1/80), respectively, while in group B the rates were 3.66% (3/82), 1.22% (1/82) and 2.44% (2/82), respectively. The incidence of major adverse cardiac events (MACEs) was not statistically different between the two groups (P>0.05), although there was a trend indicating improvement in group A (log rank χ^2^=0.953, P=0.489; Fig. 1).

## Discussion

The results of the present study suggested that the combination of a fixed dose of sodium NTP (100 *μ*g) and tirofiban, delivered via a thrombus aspiration catheter, was an effective strategy to prevent NR in patients with STEMI undergoing primary PCI. This study revealed several notable clinical implications, such as the fact that NTP plus tirofiban was more effective than tirofiban alone in improving the final CTFC, STR and TMBG of the IRA. Furthermore, the direct administration of NTP to the IRA via the thrombus aspiration catheter was generally safe and reasonably well tolerated. In addition, the peak creatine kinase (CK)-MB value was lower and the LVEF value was higher in group A than in group B. However, the incidence of MACEs was not statistically different between the two groups, although there was a trend indicating improvement in group A.

The causes of NR are complex and multifactorial. The most likely causes include platelet aggregation, distal embolization, spasm of the microcirculation, neutrophilic plugging, tissue edema or a combination of these factors (12). Prevention comprises strategies adopted prior to complete vessel re-opening, in order to prepare the microcirculation for reperfusion. Prevention strategies may be targeted to the different mechanisms of NR. Manual aspiration thrombectomy is reasonable for patients undergoing primary PCI. However, infarct size was not reduced by manual aspiration thrombectomy in the INFUSE-AMI trial of patients with large anterior STEMI (13). This may be due to the complex nature of NR, in which remote thromboembolism plays only a partial role. However, of the several mechanisms that have been proposed to explain the NR phenomenon, vasoconstriction is considered one of the most important and potentially reversible, as suggested by the numerous positive reports of therapeutic vasodilatation in this context.

NTP is a direct donor of nitric oxide (NO) and requires no intracellular metabolism to generate NO (14). It has been shown that IC NTP may lead to coronary hyperemia. Furthermore, IC NTP produces an equivalent, but more prolonged, coronary hyperemia to adenosine. Therefore, we postulated that NTP was likely to have a beneficial effect on the prevention of the NR phenomenon. However, the present study differs from previous studies in a number of respects. In previous studies, NTP was administered following the appearance of slow or no reflow, whereas in the present study the combination of NTP plus tirofiban was administered before balloon dilatation and prior to the appearance of slow or no reflow. Furthermore, in previous studies, NTP was predominantly injected into the coronary artery through the guiding catheter, whereas in the present study, NTP was injected through the thrombus aspiration catheter into the distal coronary artery to reach the occluded target lesion. The dose used in the present study is probably equivalent to a higher dose given nonselectively, as described in some of the previous studies. Therefore, the positive clinical findings of the present study were most likely due to the fact that in the clinical situation sodium NTP may alter the pathophysiology underlying the NR. NO may positively affect latent collaterals or collateral blood flow by eliciting vasodilatation or inhibiting platelet aggregation in the vascular bed distal to the target lesion (15,16). The results from the present study indicated that the delivery method used in the study was more effective than the methods used in previous studies.

The doses of NTP that have been shown to effectively treat NR have been variable, ranging from 50 to 1,000 *μ*g (7,17,18). In these previous studies, the final cumulative dose was not predefined, but was rather dictated by the ability to achieve satisfactory coronary flow and myocardial blush. We chose to use 100 *μ*g based on previous studies suggesting that doses from 0.3 to 0.9 *μ*g/kg achieved maximal coronary vasodilatation in normal coronary arteries (19). An earlier study showed that in patients with STEMI, selective IC administration of a fixed dose (60 *μ*g) of NTP failed to improve coronary flow and myocardial tissue reperfusion; however, clinical outcomes were improved at 6 months (20). The combination therapy of the PercuSurge device and 100 *μ*g NTP has been demonstrated to provide an additional benefit to NTP alone for improving microvascular circulation (9). However, that study was nonrandomized and uncontrolled. The choice of using a fixed, rather than a weight-based, dose was based upon simplicity and ease. The use of a distal injection, however, has the potential to accurately deliver medications irrespective of the proximal arterial flow.

The present study revealed positive results with regard to the epicardial flow grade and also with regard to the myocardial perfusion grade, which is thought to reflect the functionality of the coronary microvascular circulation (21). Both the TMPG and the CTFC have been shown to be associated with the extent of STR, enzymatic infarct size, nonreperfusion as defined by myocardial contrast echocardiography, left ventricular function and long-term mortality (9,22–26). It was observed in the present study that the final TMPG and CTFC in group A were significantly better than those in group B.

In the present study, selective IC NTP and tirofiban administration via a thrombus aspiration catheter was safe and well-tolerated. NTP is a potent vasodilator, and the intravenous systemic administration of NTP may sometimes markedly reduce blood pressure. However, the fixed dosage (100 *μ*g) used in this study did not result in the severe or prolonged hypotension that may have triggered a shock state.

The results of this study revealed a lower peak CK-MB value and improved TMPG, CTFC and STR trends in group A, which reflected an enhanced myocardial reperfusion and greater myocardial salvage. This may have resulted in a reduction in the incidence of composite clinical end-points. The LVEF at 6 months was higher in group A than in group B, although the incidence of MACEs was not identified to be statistically different between the two groups. However, there was a trend indicating improvement in group A. The sample size in the study may have been too small to adequately display the significance. A further study with a larger sample size and a longer follow-up period is required to further elucidate the effect of thrombus aspiration in combination with tirofiban plus NTP on the composite clinical end-points.

## Figures and Tables

**Table I.** Baseline demographic and clinical characteristics.

Variables	Group A (n=80)	Group B (n=82)	t/χ^2^	P-value
Age (years)	63±9	64±10	0.668	0.500
Male (%)	63 (78.7)	65 (79.2)	0.010	0.910
DM (%)	16 (20.0)	13 (15.9)	0.226	0.630
HTN (%)	59 (73.7)	67 (81.7)	0.959	0.330
Smokers (%)	51 (63.7)	51 (62.2)	0.010	0.972
Dyslipidemia (%)	56 (70)	52 (63.4)	0.524	0.468
Previous angina (%)	67 (83.7)	69 (84.1)	0.027	0.880
Killip class (%)			0.950	0.622
I	57 (71.3)	60 (73.2)		
II	21 (26.2)	18 (22.0)		
III	2 (2.5)	4 (4.8)		
SBP (mmHg)	135±23	129±35	1.286	0.200
DBP (mmHg)	72±8	71±9	0.747	0.450
HR (bpm)	79±20	75±17	1.373	0.170
LVEF (%)	63±9	53±11	4.679	0.001
Peak CK-MB (U/l)	170±56	210±48	4.885	0.010

DM, diabetes mellitus; HTN, hypertension; SBP, systolic blood pressure; DBP, diastolic blood pressure; HR, heart rate; LVEF, left ventricular ejection fraction; CK-MB, creatine kinase-MB.

**Table II.** Angiographic and procedural features.

Variables	Group A (n=80)	Group B (n=82)	t/χ^2^	P-value
Pain onset to PCI (h)	5.5±2.2	6.0±2.5	1.350	0.180
Door to PCI (min)	70±9	68±11	1.265	0.210
IRA			1.114	0.573
LAD	44 (55.0)	45 (54.9)		
LCX	16 (20.0)	12 (14.6)		
RCA	20 (25.0)	25 (30.5)		
Stent diameter (mm)	3.1±0.3	3.2±0.4	1.797	0.070
Stent length (mm)	21.2±6.5	20.1±7.2	1.020	0.310
Initial TIMI (%)			0.080	0.929
0	60 (75.0)	61 (74.4)		
1–2	20 (25.0)	21 (25.6)		
Final TIMI 3 (%)	74 (92.5)	75 (91.5)	0.003	0.956
Final CTFC (frames)	23±7	29±11	4.130	0.000
Final TMPG 2–3 (%)	57 (71.3)	44 (53.7)	4.351	0.030
Complete STR (%)	58 (72.5)	46 (55.9)	4.540	0.040

PCI, percutaneous coronary intervention; IRA, infarct-related artery; LAD, left anterior descending artery; LCX, left circumflex artery; RCA, right coronary artery; TIMI, thrombolysis in myocardial infarction flow grade; CTFC, corrected TIMI frame count; TMBG, TIMI myocardial perfusion grade; STR, ST-segment resolution.
